# Editorial Comments to “Overuse of Imaging in Prostate Cancer Staging”

**DOI:** 10.1111/iju.70021

**Published:** 2025-03-24

**Authors:** Yutaka Yamamoto

**Affiliations:** ^1^ Department of Urology Kindai University Nara Hospital Nara Japan

This study addresses a crucial clinical question regarding the overuse of imaging in localized prostate cancer staging [1]. This is an important issue, as the Japanese guideline does not make much reference to it. The authors analyzed real‐world data from 491 patients with localized prostate cancer, providing insights into the overuse of imaging in prostate cancer staging.

They showed that no significant metastases were detected in the low‐risk cases, and only 0.5% of the intermediate‐risk cases showed significant findings in these imaging studies. Furthermore, by eliminating computed tomography (CT) and bone scintigraphy for low‐risk prostate cancer, annual staging costs could be reduced. This study is timely and important as it also discusses the optimization of healthcare resources.

One of the notable strengths of this study is that it highlights a stark contrast between Western clinical guidelines and real‐world practice in Japan. While the National Comprehensive Cancer Network (NCCN) guideline recommends omitting CT and bone scintigraphy for low‐risk prostate cancer [2], this study reveals that 88.3% and 67.5% of low‐risk patients still undergo these imaging procedures. These findings underline the persistence of outdated practices in Japan, despite evolving recommendations in the United States. Furthermore, the economic analysis in this study is also commendable. By estimating the potential cost savings of omitting unnecessary imaging in low‐risk patients, it provides a tangible measure of the financial burden of overuse. The reported potential annual savings of USD 4.07 million (JPY 607 million) highlight the broader economic implications of aligning clinical practices with guidelines.

Despite its strengths, some limitations should be acknowledged. First, this study primarily focuses on conventional imaging modalities (CT and bone scintigraphy) without discussing the role of emerging imaging technologies such as prostate‐specific membrane antigen positron emission tomography (PSMA‐PET) or whole‐body MRI. These newer modalities have demonstrated superior sensitivity and specificity for detecting metastatic prostate cancer [3], and are increasingly being incorporated into international guidelines [4]. A discussion on how these newer modalities may influence future staging practices would strengthen this study's implications.

Additionally, while the study suggests that omitting unnecessary imaging could lead to cost savings, it does not address the potential benefits of incidental findings. The authors note that 2.5% of cases revealed incidental findings, some of which were clinically significant. Previous studies suggest that incidental malignancies detected during prostate cancer staging can range from 1.2% to 2% [5]. The balance between cost‐effectiveness and the potential benefits of early detection of other malignancies warrants further discussion.

Overall, this study provides valuable insights into the persistent overuse of imaging in prostate cancer staging in Japan. The findings emphasize the need for ongoing education and systemic interventions to align clinical practice with evidence‐based guidelines. Future research should explore the integration of advanced imaging techniques and assess the long‐term clinical outcomes associated with imaging omission in low‐risk patients.

## Author Contributions

None.

## Conflicts of Interest

The author declares no conflicts of interest.
